# Neurobiological and behavioral relevance of intrinsic functional connectome constraints on task-evoked neural activation

**DOI:** 10.1016/j.isci.2026.116903

**Published:** 2026-07-22

**Authors:** Chao Tao, Shunshun Cui, Yuhao Shen, Yan Cheng, Jiajia Zhu, Yongqiang Yu

**Affiliations:** 1 Department of Radiology, The First Affiliated Hospital of Anhui Medical University, Hefei 230022, China; 2 Research Center of Clinical Medical Imaging, Hefei, Anhui Province 230032, China; 3 Anhui Provincial Key Laboratory for Brain Bank Construction and Resource Utilization, Hefei 230032, China

**Keywords:** resting-state functional connectivity, task-evoked neural activation, brain connectome, functional magnetic resonance imaging, cognition

## Abstract

Elucidating how resting-state functional connectivity relates to task-evoked neural activation is an important topic in systems neuroscience. We analyzed task-based and resting-state functional magnetic resonance imaging data from 1,005 participants from the Human Connectome Project. On the basis of connectome-constrained predictive modeling, we calculated a neural activation constraint index (NACI) to assess the extent to which intrinsic functional connectome architecture constrains task-evoked neural activation. NACIs showed task-dependent variations, indicating differential constraint effects of the intrinsic functional connectome across distinct tasks. Spectral clustering based on the NACI classified participants into the high- and low-constraint groups. The high-constraint group exhibited superior cognitive functions in several domains and better performance across multiple tasks. Higher NACIs from working memory, language, and relational tasks correlated with greater cognitive functions and better task performance. These findings support the neurobiological and behavioral relevance of NACI and suggest its utility for characterizing individual differences in functional brain organization.

## Highlights

• Intrinsic connectome shapes task-evoked neural activation patterns
• NACI captures how connectivity constrains brain activation across tasks
• Higher NACI is linked to better cognition across multiple domains
• NACI predicts task accuracy and faster behavioral responses

## Graphical abstract

**Figure:**
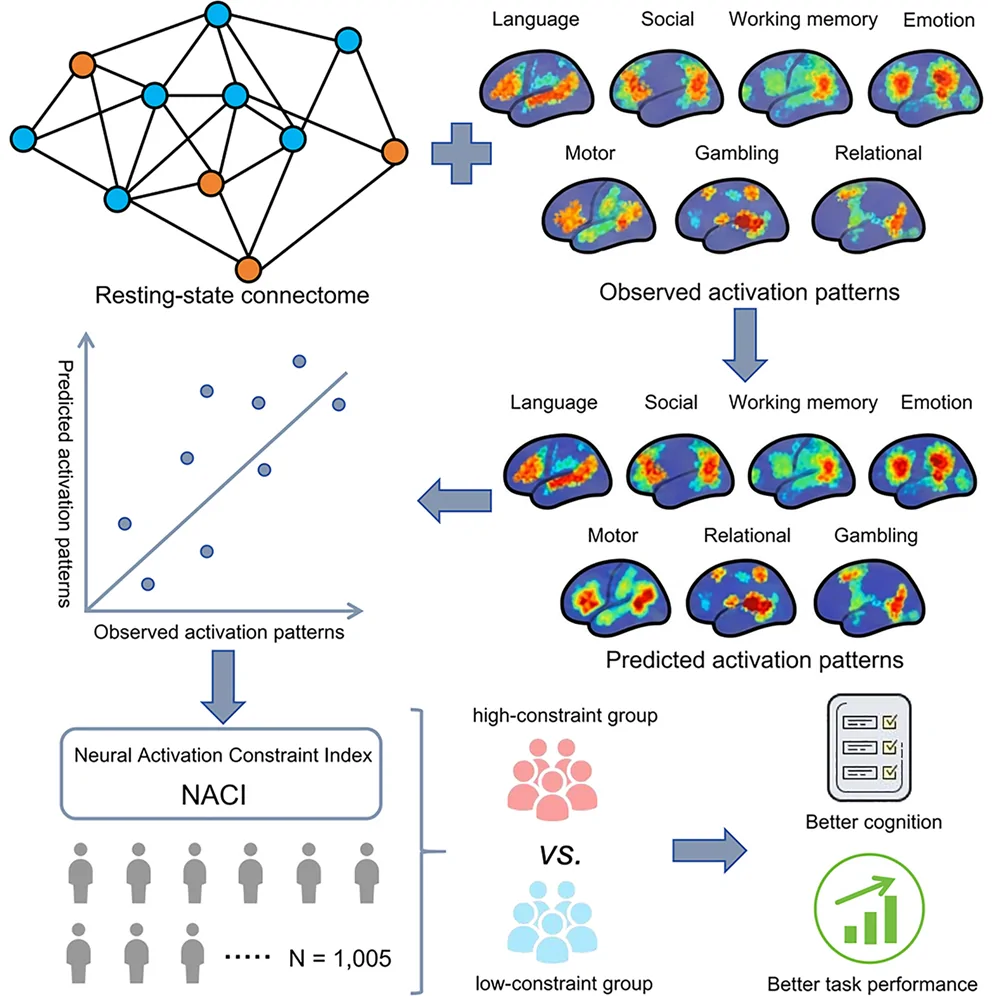

## Teaser

Neuroscience; cognitive neuroscience

## Introduction

Functional neuroimaging evidence demonstrates that a certain stimulation or task could elicit responses in a specific set of spatially distributed brain areas, referred to as task-evoked neural activation. Different task paradigms involving the cognitive, affective, and sensorimotor domains induce distinct neural activation patterns. These patterns reflect task-dependent neurobiological processes underlying information processing.^1^^,^^2^^,^^3^^,^^4^ The brain also remains intrinsically active in the absence of explicit tasks or stimuli, giving rise to a stable functional architecture during rest. Resting-state functional connectivity (rsFC), typically quantified as temporal correlations of spontaneous neural activity between regions, has been widely used to characterize this intrinsic organization.^5^^,^^6^^,^^7^ Elucidating how rsFC relates to task-evoked neural activation is an important topic in systems neuroscience.

Accumulating evidence suggests that the intrinsic functional network architecture shapes task-evoked activation patterns and may provide a functional scaffold for task processing.^8^^,^^9^^,^^10^ Consistent with this view, prior studies have shown that rsFC can predict task-evoked activation patterns at both group and individual levels, including approaches based on regression or machine learning,^11^^,^^12^^,^^13^ as well as whole-brain predictive mapping frameworks.^14^ This line of research is conceptually related to the activity flow modeling framework, which proposes that intrinsic functional connectivity can mediate the propagation of neural activity across regions to predict task-evoked activation patterns.^15^ In addition, prior work has examined regional variability in the activity flow prediction accuracy, which is conceptually related to the present study.^16^ However, existing approaches primarily focus on the prediction accuracy and often lack interpretability at the individual level. In particular, it remains unclear to what extent the intrinsic functional connectome architecture constrains task-evoked neural activation within individuals.

**Figure 1:**
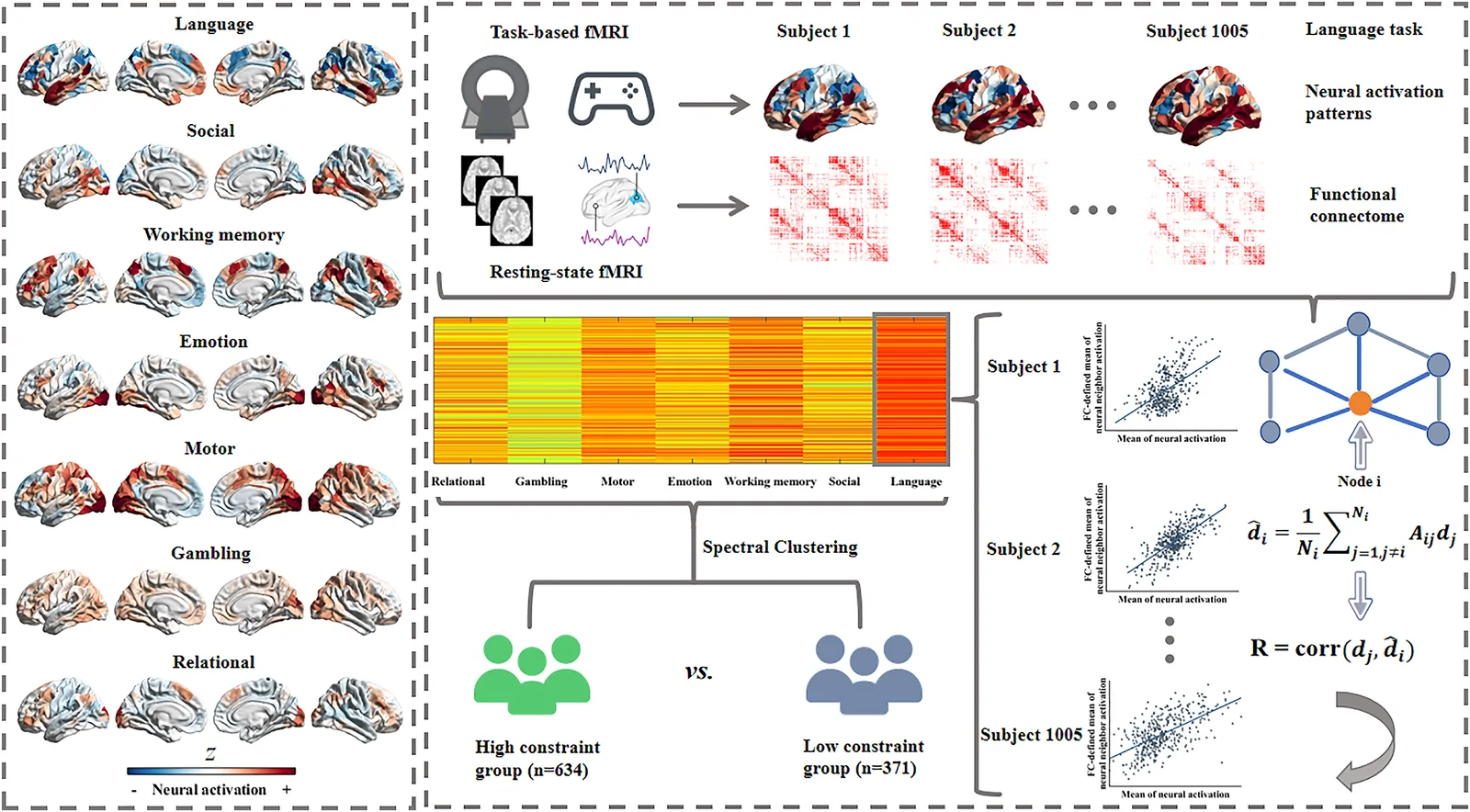
Research design and analytical pipeline We conducted a combined analysis of tb-fMRI and rs-fMRI data of 1,005 participants from the HCP dataset. The tb-fMRI data were used to measure individual neural activation patterns during a range of cognitive, affective, and sensorimotor tasks. The rs-fMRI data were utilized to construct individual functional connectomes. Based on an updated connectome-constrained predictive modeling, we calculated a NACI to assess the extent to which the intrinsic functional connectome architecture constrains task-evoked neural activation at the individual level. Task-dependent variations in NACI were then investigated. To further explore their neurobiological and behavioral relevance, we (1) combined the NACI features with spectral clustering to classify the participants into different groups followed by between-group comparisons in demographic and behavioral characteristics, and (2) directly examined the associations of NACI with cognitive functions and task performance. Abbreviations: tb-fMRI, task-based functional magnetic resonance imaging; rs-fMRI, resting-state functional magnetic resonance imaging; HCP, Human Connectome Project; NACI, Neural Activation Constraint Index.

In this study, we conducted a combined analysis of task-based and resting-state functional magnetic resonance imaging (tb-fMRI and rs-fMRI, respectively) data from 1,005 participants in the Human Connectome Project (HCP) dataset. The tb-fMRI data were used to measure individual neural activation patterns across a variety of cognitive, affective, and sensorimotor tasks, while the rs-fMRI data were utilized to construct individual functional connectomes. To quantify the extent to which the intrinsic functional connectome architecture constrains task-evoked activations, we developed an interpretable metric termed the neural activation constraint index (NACI). Unlike prior approaches that primarily emphasize prediction accuracy, NACI is specifically designed to quantify the strength of connectome-imposed constraints at the individual level, providing a complementary perspective on connectome-activation relationships. This index is conceptually grounded in the principle that task-evoked activations are shaped by activity propagation through the intrinsic connectome. Task-dependent variations in NACI were then investigated. To further explore their neurobiological and behavioral relevance, we (1) combined the NACI features with spectral clustering to classify the participants into different groups followed by between-group comparisons in demographic and behavioral characteristics, and (2) directly examined the associations of NACI with cognitive functions and task performance. A schematic of the research design and analytical pipeline is shown in Figure 1.

## Results

### Task-dependent variations in NACIs

**Figure 2:**
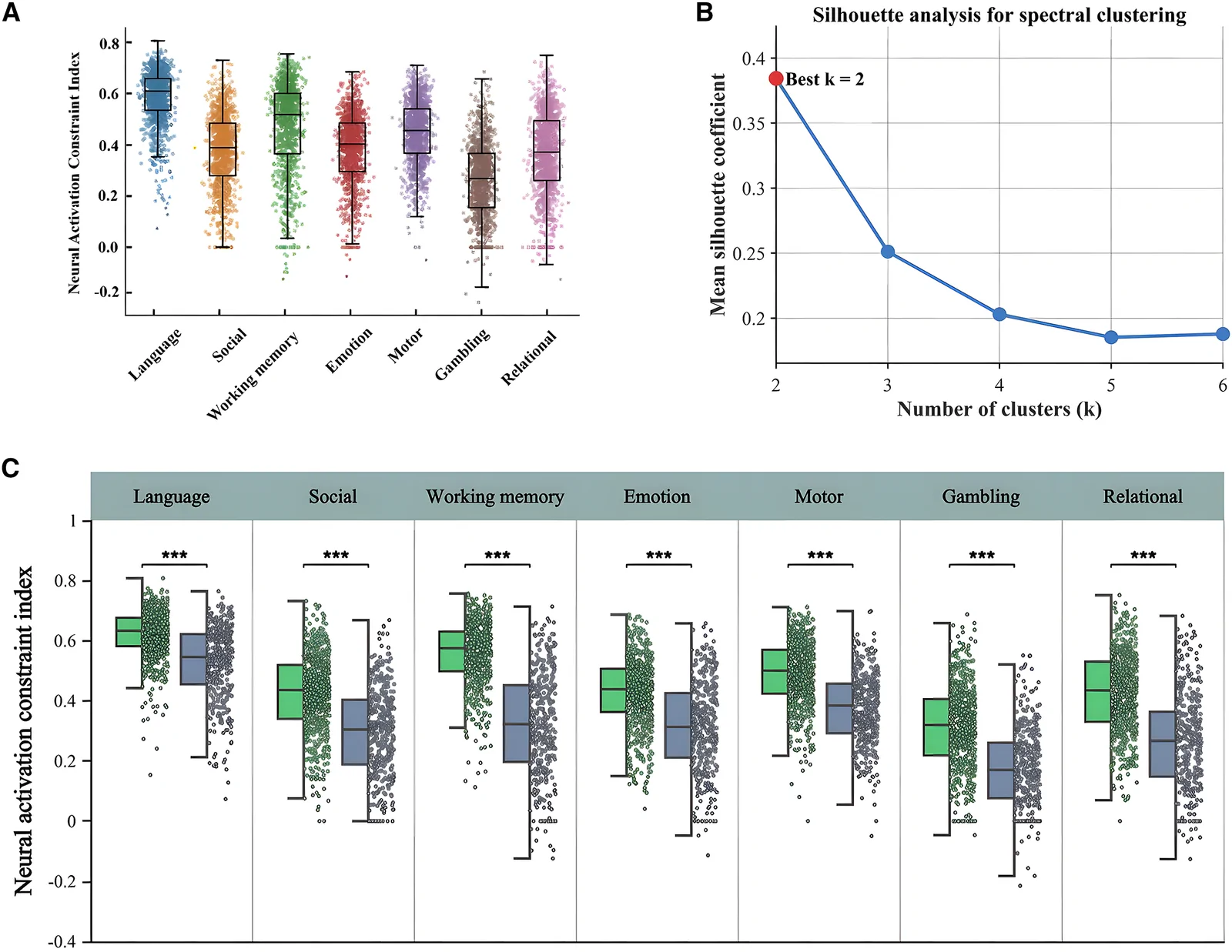
Task-dependent variations in NACI and NACI-based grouping (A) Boxplots showing the median, interquartile range, and overall distribution of NACI values from the seven tasks. (B) Mean Silhouette coefficients were calculated across candidate cluster numbers (*K* = 2–6). The highest mean Silhouette coefficient was observed at *K* = 2 (0.384). (C) Boxplots showing group differences in NACI across the seven tasks. Statistical significance is indicated by asterisks: ∗*p*-FDR < 0.05; ∗∗*p*-FDR < 0.01; and ∗∗∗*p*-FDR < 0.001. Abbreviations: NACI, Neural Activation Constraint Index; PC, principal component; FDR, false discovery rate.

As shown in Figure 2A, there were significant differences in NACIs across the seven task paradigms (*F* = 619.05, *p* < 0.001). Post hoc analyses revealed a pattern of task-dependent variations in NACIs: language (0.587 ± 0.108) > working memory (0.470 ± 0.177) > motor (0.448 ± 0.125) > emotion (0.385 ± 0.140) > social (0.377 ± 0.150) > relational (0.367 ± 0.165) > gambling (0.262 ± 0.151), with almost all paired comparison results being statistically significant (*p* < 0.05, false discovery rate [FDR] corrected) except for relational vs. social (*p*-FDR = 0.122) and social vs. emotion (*p*-FDR = 0.143). Detailed information on the results of the paired comparisons is provided in Table S1.

### NACI-based grouping

Spectral clustering based on the NACI features classified the 1,005 subjects into two groups. The number of clusters was determined by evaluating the mean Silhouette coefficient across a range of cluster numbers (*K* = 2–6), with the highest value observed at *K* = 2 (0.384), indicating the optimal clustering solution (Figure 2B). As illustrated in Figure 2C, the group of 634 subjects showed consistently higher NACI values across the seven task paradigms than the other group of 371 subjects (language: 0.621 ± 0.080 vs. 0.527 ± 0.123, *t* = 14.649, *p*-FDR < 0.001; social: 0.423 ± 0.130 vs. 0.296 ± 0.147, *t* = 14.238, *p*-FDR < 0.001; working memory: 0.557 ± 0.106 vs. 0.322 ± 0.176, *t* = 26.408, *p*-FDR < 0.001; emotion: 0.427 ± 0.114 vs. 0.313 ± 0.151, *t* = 13.530, *p*-FDR < 0.001; motor: 0.492 ± 0.106 vs. 0.374 ± 0.119, *t* = 16.215, *p*-FDR < 0.001; gambling: 0.311 ± 0.138 vs. 0.177 ± 0.134, *t* = 14.981, *p*-FDR < 0.001; and relational: 0.427 ± 0.133 vs. 0.263 ± 0.163, *t* = 17.312, *p*-FDR < 0.001), such that the former was defined as the high-constraint group and the latter as the low-constraint group.

With respect to demographic variables, the high-constraint group was preferentially female (χ^2^ = 11.714, *p* = 0.001), younger (*t* = −2.193, *p* = 0.029), more highly educated (*t* = 4.116, *p* < 0.001), and had a higher income level (*t* = 2.348, *p* = 0.019) compared with its low-constraint counterpart. In addition, the high-constraint group showed lower head motion, as indexed by framewise displacement (FD) (*t* = −5.391, *p* < 0.001) (Table 1). No significant differences were found in the body mass index (BMI), handedness, Pittsburgh sleep quality index (PSQI) total score, and family psychiatric history (*p* > 0.05).

**Table 1:** Demographic characteristics of the high- and low-constraint groups

	High-constraint group (*n* = 634)	Low-constraint group (*n* = 371)	*t*/*χ*^*2*^	*p* value
Female, *n* (%)	363 (57.3%)	171 (46.1%)	11.714	0.001
Age, years, mean (SD)	28.53 (3.69)	29.06 (3.71)	-2.193	0.029
BMI, kg/m^2^, mean (SD)	26.20 (4.90)	26.70 (5.29)	-1.500	0.134
Right-handed, *n* (%)	581 (91.6%)	331(89.2%)	1.635	0.201
Education, years, mean (SD)	15.15 (1.66)	14.65 (1.93)	4.116	<0.001
Income, USD/year, mean (SD)	5.21 (2.12)	4.89 (2.15)	2.348	0.019
PSQI total score, mean (SD)	4.62 (2.70)	4.92 (2.82)	-1.643	0.101
Family psychiatric history, *n* (%)	114 (18.0%)	73 (19.7%)	-0.444	0.505
FD, mm, mean (SD)	0.083 (0.03)	0.094 (0.03)	-5.391	<0.001

BMI, body mass index; FD, Framewise displacement; PSQI, Pittsburgh sleep quality index; SD, standard deviation.

### Group differences in cognitive functions and task performance

**Figure 3:**
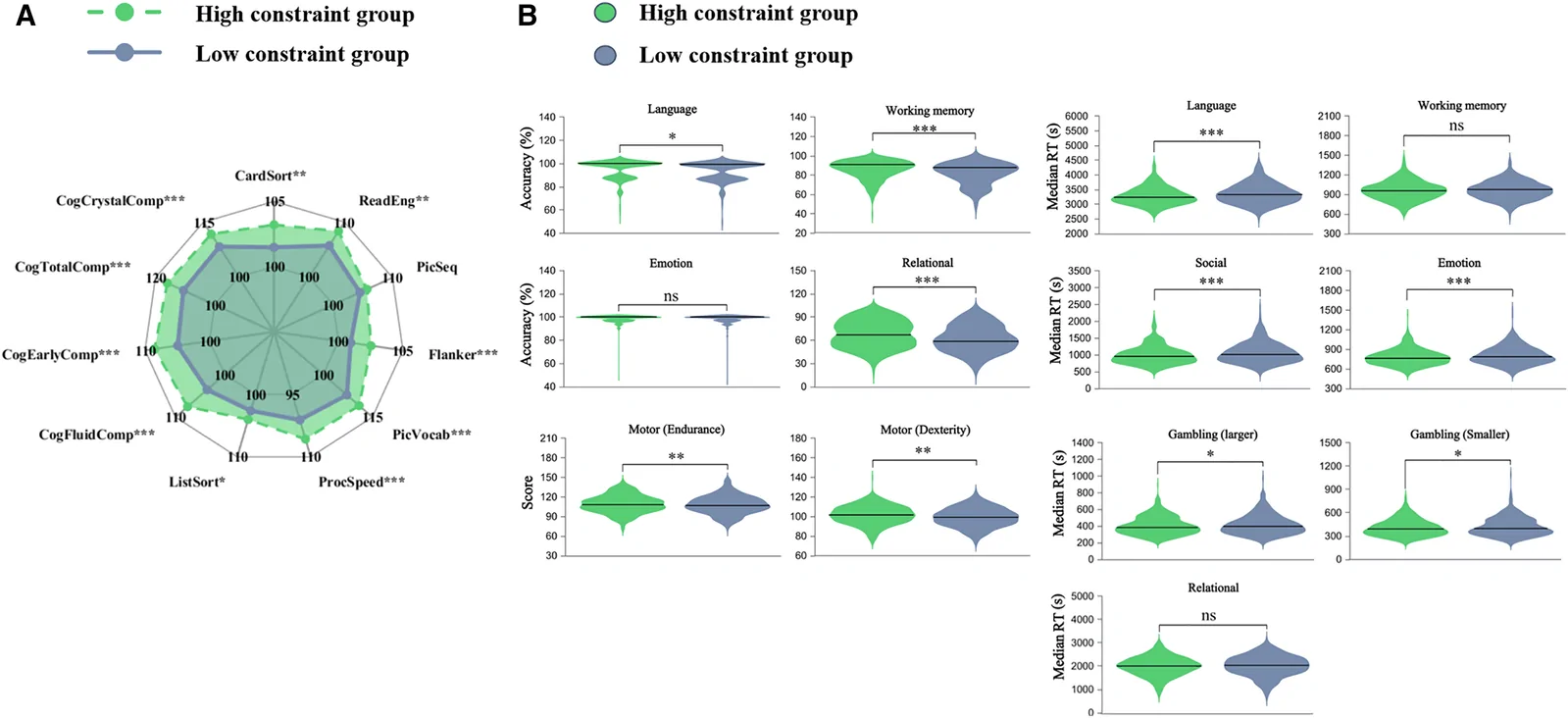
Differences in cognitive functions and task performance between the high- and low-constraint groups (A) Radar plot showing group differences in cognitive functions across multiple domains. (B) Violin plots illustrating group differences in task performance across the seven task paradigms. Statistical significance is denoted by asterisks: ∗*p*-FDR < 0.05; ∗∗*p*-FDR < 0.01; and ∗∗∗*p*-FDR < 0.001. Abbreviations: CardSort, dimensional change card sort; ReadEng, oral reading recognition; PicSeq, picture sequence memory; Flanker, flanker inhibitory control and attention; PicVocab, picture vocabulary; ProcSpeed, pattern comparison processing speed; ListSort, list sorting working memory; CogFluidComp, cognitive fluid intelligence composite; CogEarlyComp, cognitive early childhood composite; CogTotalComp, cognitive total composite; CogCrystalComp, cognitive crystallized intelligence composite; RT, reaction time; ns, non-significant.

Compared with the low-constraint group, the high-constraint group exhibited better cognitive functions in many domains, i.e., showing higher scores in the dimensional change card sort test (CardSort), oral reading recognition test (ReadEng), flanker inhibitory control and attention test (Flanker), picture vocabulary test (PicVocab), pattern comparison processing speed test (ProcSpeed), list-sorting working memory test (ListSort), cognitive fluid intelligence composite (CogFluidComp), cognitive crystallized intelligence composite (CogCrystalComp), cognitive total composite (CogTotalComp), and cognitive early childhood composite (CogEarlyComp) (*p* < 0.05, FDR corrected) (Figure 3A; Table S2). In addition, the high-constraint group achieved better task performance across multiple task paradigms than the low-constraint group (Figure 3B; Table S2). Specifically, the high-constraint group had higher accuracy in language, working memory, and relational tasks, as well as shorter reaction times in language, working memory, emotion, and social and gambling tasks (*p* < 0.05, FDR corrected). For motor task, the high-constraint group outperformed its low-constraint counterpart in terms of endurance and dexterity (*p* < 0.05, FDR corrected).

### Associations of NACI with cognitive functions and task performance

**Figure 4:**
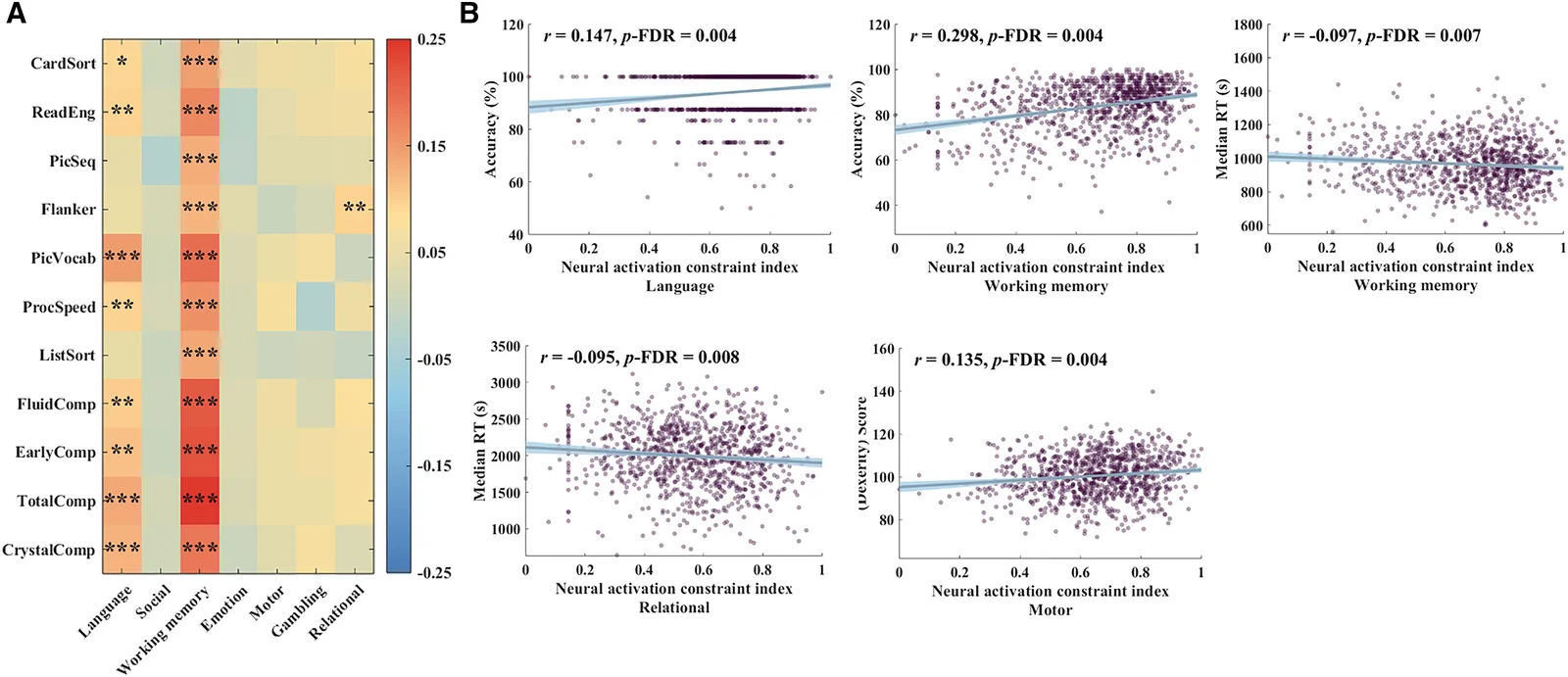
Associations of NACI with cognitive functions and task performance (A) Heatmap showing the correlations between NACIs from the seven task paradigms and cognitive functions across multiple domains. Color bar represents correlation coefficients. (B) Scatterplots displaying the significant correlations between NACIs from several task paradigms and their corresponding task performance. Statistical significance is denoted by asterisks: ∗*p*-FDR < 0.05; ∗∗*p*-FDR < 0.01; and ∗∗∗*p*-FDR < 0.001. Abbreviations: CardSort, dimensional change card sort; ReadEng, oral reading recognition; PicSeq, picture sequence memory; Flanker, flanker inhibitory control and attention; PicVocab, picture vocabulary; ProcSpeed, pattern comparison processing speed; ListSort, list-sorting working memory; FluidComp, cognitive fluid intelligence composite; EarlyComp, cognitive early childhood composite; TotalComp, cognitive total composite; CrystalComp, cognitive crystallized intelligence composite; FDR, false discovery rate.

As shown in Figure 4A and Table S4, NACIs from working memory, language, and relational tasks were positively correlated with cognitive functions, with NACI from the working memory task showing the strongest and widest associations. Specifically, NACI from the working memory task was positively correlated with CardSort, ReadEng, PicSeq, Flanker, PicVocab, ProcSpeed, ListSort, CogFluidComp, CogCrystalComp, CogTotalComp, and CogEarlyComp (*p* < 0.05, FDR corrected). NACI from language task was positively correlated with CardSort, ReadEng, PicVocab, ProcSpeed, CogFluidComp, CogCrystalComp, CogTotalComp, and CogEarlyComp (*p* < 0.05, FDR corrected). NACI from the relational task was positively correlated with flanker (*p* < 0.05, FDR corrected). There were no significant associations between cognitive functions and NACI from social, emotion, motor, or gambling tasks (*p* > 0.05, FDR corrected).

The associations between NACI from the seven task paradigms and their corresponding task performance are displayed in Figures 4B and S1. For language task, NACI was positively correlated with accuracy (*r* = 0.147, *p*-FDR = 0.004) but was not significantly correlated with reaction time. For working memory task, NACI was positively correlated with accuracy (*r* = 0.298, *p*-FDR = 0.004) and negatively correlated with reaction time (*r* = −0.097, *p*-FDR = 0.007). For motor task, NACI was positively correlated with dexterity (*r* = 0.135, *p*-FDR = 0.004) but was not significantly correlated with endurance. For relational task, NACI was negatively correlated with reaction time (*r* = −0.095, *p*-FDR = 0.008) but was not significantly correlated with accuracy. For social, emotion, or gambling tasks, however, there were no significant correlations between NACI and task performance (*p* > 0.05, FDR corrected).

### Sensitivity analyses controlling for potential confounds

To assess whether the observed associations between NACI and behavioral performance were driven by potential confounding factors, we conducted sensitivity analyses, controlling for age, sex, head motion, education, and income. Compared with the base model (controlling for age and sex), the inclusion of additional covariates attenuated the strength of several associations. However, multiple key effects remained significant after FDR correction, including working memory accuracy (2-back), relational task accuracy, and reaction time measures in the language, social, and emotion tasks (Table S3).

### Robustness of NACI across analysis pipelines

Across all robustness analyses, spectral clustering of the recalculated NACI vectors consistently supported a two-cluster solution, with the highest Silhouette coefficient observed at *K* = 2 under each condition (Figure S2). NACI values showed moderate-to-high consistency when global signal regression was applied (Pearson’s *r* = 0.64–0.81; consistency = 74.2%; Cohen’s *κ* = 0.47) and high consistency for the positive-only connectivity condition (*r* = 0.95–0.98; consistency = 94.0%; *κ* = 0.87), HCP multimodal parcellation (Glasser-360) (*r* = 0.85–0.92; consistency = 89.7%; *κ* = 0.78), and the connectivity threshold condition (Functional Connectivity [FC] > 0.1; *r* = 0.91–0.94; consistency = 91.6%; *κ* = 0.82) (Table S5).

## Discussion

Based on large-scale tb-fMRI and rs-fMRI data from the HCP, we propose the NACI to assess the extent to which intrinsic functional connectome architecture constrains task-evoked neural activation at the individual level. Three main findings were observed in this study. First, there were task-dependent variations in NACI, suggesting differential constraint effects of the intrinsic functional connectome on neural activation profiles across distinct tasks. Second, individuals with higher NACI exhibited better cognitive functions in many domains and achieved better task performance across multiple task paradigms relative to those with lower NACI. Third, higher NACIs from the working memory, language, and relational tasks were associated with better cognitive functions, with NACI from the working memory task showing the strongest and widest associations; in addition, higher NACIs from the working memory, language, motor, and relational tasks were related to better performance in the corresponding tasks. After controlling for age, sex, head motion, education, and income, associations with broad cognitive composite scores were substantially attenuated, whereas associations with selected task-performance measures—particularly reaction times in the language, social, and emotion tasks, as well as accuracy in working memory 2-back and relational tasks—remained statistically significant. Notably, most of the associations that remained significant after full covariate adjustment involved reaction-time measures. Associations with accuracy and broad cognitive composite scores were generally more strongly attenuated, although the associations with working memory 2-back accuracy and relational task accuracy remained significant. This pattern may reflect the nature of what NACI captures: the constraint of intrinsic connectivity on activation propagation may be most directly manifested in the speed of neural information transmission, consistent with the recognized role of processing speed as a fundamental component of cognitive ability.^17^^,^^18^ However, we cannot rule out that the residual behavioral relevance of NACI is driven largely by general cognitive speed, rather than domain-specific connectivity constraints. Future studies with tasks placing greater demands on domain-specific processing may help disentangle these interpretations. Together, these findings support the neurobiological and behavioral relevance of NACI as an individual-level measure of FC-to-activation correspondence.

Resting-state functional connectome reflects the brain’s intrinsic organization. Its topology shows high inter-individual stability and is associated with cognitive abilities.^19^^,^^20^^,^^21^ Previous studies have used rsFC to predict task-related neural activation, but they are limited by coarse spatial resolution and high task variability.^8^^,^^9^^,^^14^^,^^22^^,^^23^^,^^24^ Conceptually grounded in the principle that task-evoked activations are shaped by activity propagation through the intrinsic functional connectome, consistent with the activity flow modeling framework, we propose NACI to quantify the extent to which the intrinsic functional connectivity constrains task-evoked neural activation at the individual level. The current observation of high NACIs across most tasks supports the idea that resting-state functional connectome indeed constrains and shapes the task-evoked neural activity. Furthermore, we found task-dependent variations in NACI. That said, NACIs from the language and working memory tasks were higher than those from the emotion and gambling tasks. This suggests that the tasks with high cognitive load, such as language and working memory, rely more on well-organized intrinsic connectivity pathways.^25^^,^^26^ These tasks may depend less on individual strategies during task execution. In contrast, emotional processing and gambling decision-making tasks are more likely to be influenced by the emotional valence and motivational factors, with their neural processing modulated by real-time environmental and individual endogenous states, rather than primarily relying on the intrinsic architecture of rsFC network.^27^^,^^28^ This view aligns with Shine and Mohr et al.’s research, which indicated that the brain flexibly reconfigures the cooperation patterns between brain regions based on current task demands, a process dynamically regulated by the neural modulation system.^29^^,^^30^

After establishing a significant association between the NACI and cognition, we further elucidated its theoretical implications in neural regulation. Variability in NACI across individuals may reflect both the efficiency of rsFC and the possible compensatory mechanisms. We observed that individuals with higher NACIs tended to perform better on multidimensional tasks, such as achieving higher scores, greater accuracy, and shorter reaction times. This improved performance may reflect a more efficient intrinsic connectivity architecture during rest. Such an organization may facilitate rapid and stable recruitment of task-relevant neural resources. This mechanism enables the brain to quickly transition into a high-efficiency mode when engaging in tasks, rather than merely amplifying the activation intensity. These findings provide unique evidence supporting the neural efficiency hypothesis,^31^^,^^32^^,^^33^^,^^34^ suggesting that superior cognitive performance relies on a finely tuned functional network preconfiguration established during the resting state. This preconfiguration optimizes neural energy metabolism and information transmission efficiency, ultimately ensuring the optimal allocation and utilization of cognitive resources. In addition to these continuous brain-behavior associations, the clustering analysis provided a data-driven summary of individual variability in NACI profiles. However, the resulting groups should be interpreted cautiously. Given that the NACI values were correlated across tasks, the high- and low-constraint groups can be more appropriately viewed as a stratification along an overall FC-to-activation correspondence dimension, rather than as discrete biological subtypes. Regarding the robustness of NACIs, classification consistency was high under the positive-only connectivity (κ = 0.87), alternative parcellation (κ = 0.78), and connectivity threshold (FC > 0.1; κ = 0.82) conditions but relatively low under global signal regression (GSR; κ = 0.47). This discrepancy likely reflects GSR’s well-documented effects on the connectivity estimates, including the introduction of artifactual negative correlations and the removal of neurophysiologically meaningful global signal variance.^35^^,^^36^ Nevertheless, the correlation between GSR-based and original NACI values remained moderate to high (r = 0.64–0.81), suggesting that the classification discrepancies were concentrated among subjects near the group boundary. The sensitivity of NACI to GSR represents a limitation, consistent with the broader ongoing debate regarding the appropriateness of GSR in functional connectivity analyses.^37^

Cognitive impairment is a core feature of severe mental illnesses such as schizophrenia and bipolar disorder and is also an early clinical manifestation of neurodegenerative diseases like Alzheimer disease.^38^^,^^39^^,^^40^^,^^41^ Current research shows significant differences in the neural mechanisms of cognitive impairment across these two categories: mental illnesses are linked to prefrontal-limbic network dysregulation, as well as dysfunction in the executive control network,^42^^,^^43^ while the latter, such as Alzheimer disease, may involve a dissociation between the default mode network and the hippocampal system.^44^^,^^45^ This mechanistic divergence makes it difficult for the existing diagnostic tools to capture shared features of cognitive deficits across disorders. As a result, early detection and intervention remain challenging. NACI, measuring how rsFC influences task-related neural activation, was found to correlate with multidimensional cognitive abilities and predict the cognitive task performance, offering a unified framework for understanding individual cognitive differences. The ability to reflect cross-state brain functional integration suggests that NACI may provide a useful framework for characterizing individual differences in cognition. However, its potential clinical relevance remains speculative and requires validation in independent clinical cohorts.

In conclusion, our work introduced NACI, an individual-level metric that quantifies the extent to which the intrinsic functional connectome architecture constrains task-evoked neural activation. NACI exhibited task specificity and broad associations with cognitive functions and task performance, which support its neurobiological and behavioral relevance. More generally, NACI may provide a useful framework for characterizing individual differences in the relationship between intrinsic functional connectivity and task-evoked neural activation.

### Limitations of the study

There are several limitations of the current study that we need to acknowledge. First, our study sample consisted solely of healthy young adults aged 22–35 years, which may limit the generalizability of the findings. Future research should include participants across a broader age range and clinical populations to assess the broad utility of NACI. Second, the computation of NACI relies on static functional connectivity and does not consider the temporally dynamic reconfiguration of brain networks during task execution. Using a dynamic functional connectome with elaborate spatiotemporal dynamics at multiple timescales may more accurately capture neural adaptations under tasks. Third, the estimation of functional connectivity in this study was based on Pearson’s correlation, which may be influenced by indirect connections and global signal effects. Although our results were robust across different preprocessing strategies and connectivity definitions, more advanced approaches such as regularized partial correlation methods may provide improved estimates of direct functional interactions.^46^ Incorporating these methods in future work may further refine the characterization of connectivity constraints. Fourth, the number of task paradigms used in the current study is relatively limited. Although the seven tasks in the HCP dataset span multiple cognitive, affective, and sensorimotor domains, they may not fully capture the diversity of brain functional states. Recent large-scale datasets with more extensive multitask batteries provide valuable opportunities to extend the present framework.^47^^,^^48^ Incorporating a broader range of task conditions may enable a more comprehensive characterization of individual differences in connectivity constraints. Fifth, the predictive model did not consider important topological properties, such as clustering and path length, which reflect local specialization and global integration, respectively, of brain function. Incorporating multilevel network characteristics in future studies may enhance prediction accuracy for complex tasks. Finally, head motion is not merely a source of artifact but may itself have a neurobiological basis, as individual differences in head motion have been shown to reflect stable trait-like neural characteristics associated with distinct functional connectivity patterns.^49^ Thus, statistically controlling for head motion could inadvertently remove neurobiologically meaningful variance, and motion-corrected results should be interpreted with this caveat in mind.

## Resource availability

### Lead contact

Requests for further information and resources should be directed to and will be fulfilled by the lead contact, Yongqiang Yu (yuyongqiang@ahmu.edu.cn).

### Materials availability

This study did not generate new materials.

### Data and code availability

• This study uses publicly available data from the HCP, which can be accessed at https://www.humanconnectome.org/.
• The MATLAB code used for NACI calculation, spectral clustering, robustness analyses, and cluster consistency analyses is publicly available at https://github.com/1421828675-arch/NACI-spectral-clustering-HCP.
• Any additional information required to reanalyze the data reported in this paper is available from the lead contact upon reasonable request.

## Acknowledgments

We would like to thank all the participants. This work was supported by the 10.13039/501100001809National Natural Science Foundation of China (grant numbers: 82471952 and 82371928), the STI2030-Major Projects (grant number: 2022ZD0205200), the 10.13039/501100003995Anhui Provincial Natural Science Foundation (grant number: 2308085MH277), the Scientific Research Key Project of Anhui Province Universities (grant number: 2022AH051135), and the Scientific Research Foundation of Anhui Medical University (grant number: 2022xkj143).

## Author contributions

Conceptualization, C.T., J.Z., and Y.Y.; methodology, C.T., S.C., Y.S., Y.C., J.Z., and Y.Y.; investigation, C.T., J.Z., and Y.Y.; visualization, C.T.; writing – original draft, C.T.; writing – review & editing, J.Z. and Y.Y.; supervision, J.Z. and Y.Y.; funding acquisition, J.Z. and Y.Y.

## Declaration of interests

The authors declare no competing interests.

## STAR★Methods

### Key resources table

**Table:** 

REAGENT or RESOURCE	SOURCE	IDENTIFIER
**Deposited data**		

Human Connectome Project (HCP) S1200 dataset	Van Essen et al.^50^	https://www.humanconnectome.org/study/hcp-young-adult
NIH Toolbox Cognition Battery	Weintraub et al.^51^	https://www.nihtoolbox.org/

**Software and algorithms**		

MATLAB R2021a	MathWorks	https://www.mathworks.com/
SPSS Statistics v26.0	IBM	https://www.ibm.com/spss
R (v4.1.0)	R Foundation	https://www.r-project.org/
Python (v3.8)	Python Software Foundation	https://www.python.org/
DPABI toolbox	Yan et al.^52^	http://rfmri.org/dpabi
NACI calculation and spectral-clustering code	This paper	GitHub repository: https://github.com/1421828675-arch/NACI-spectral-clustering-HCP

**Other**		

Schaefer 400-parcel cortical atlas	Schaefer et al.^53^	https://github.com/ThomasYeoLab/CBIG
HCP multimodal parcellation (Glasser-360)	Glasser et al.^54^	https://balsa.wustl.edu/study/show/RVVG
HCP preprocessing pipeline	Glasser et al.^55^	https://github.com/Washington-University/HCPpipelines

### Experimental model and study participant details

#### Human participants

The data used in this study were obtained from the publicly available S1200 release of the Human Connectome Project (HCP) database (https://www.humanconnectome.org/study/hcp-young-adult).^50^ A total of 1,005 healthy young adults aged 22 to 35 years (471 males) were included in the analyses. All participants were screened according to HCP protocols and reported no history of neurological or psychiatric disorders.

All participants provided written informed consent, and the study was approved by the Washington University Institutional Review Board in accordance with relevant guidelines and regulations.

#### Demographic and behavioral variables

Demographic variables included sex, age, body mass index (BMI), handedness, years of education, annual household income, Pittsburgh Sleep Quality Index (PSQI), framewise displacement (FD), and family history of psychiatric disorders.

#### Cognitive assessment

The NIH Toolbox Cognition Battery was employed to assess cognitive functions across a range of domains and compute composite scores, including Dimensional Change Card Sort Test (CardSort), Oral Reading Recognition Test (ReadEng), Picture Sequence Memory Test (PicSeq), Flanker Inhibitory Control and Attention Test (Flanker), Picture Vocabulary Test (PicVocab), Pattern Comparison Processing Speed Test (ProcSpeed), List Sorting Working Memory Test (ListSort), Cognitive Fluid Intelligence Composite (CogFluidComp), Cognitive Early Childhood Composite (CogEarlyComp), Cognitive Total Composite (CogTotalComp), and Cognitive Crystallized Intelligence Composite (CogCrystalComp), with all scores adjusted for age.

#### Task performance

Task performance across the seven task paradigms was assessed with the following key outcome measures: 1) Language: accuracy in story comprehension (Story Acc) and median reaction time (Story Median RT); 2) Social: median reaction time for Theory of Mind (Median RT ToM); 3) Working memory: accuracy in the 2-back task (2bk Acc) and corresponding median reaction time (2bk Median RT); 4) Emotion: accuracy in facial emotion recognition (Face Acc) and median reaction time (Face Median RT); 5) Motor: performance in physical endurance and manual dexterity (Endurance and Dexterity); 6) Gambling: median reaction time in risk-based decision-making (Median RT Larger/Smaller); and 7) Relational: accuracy in relational inference (Rel Acc) and median reaction time (Rel Median RT).

### Method details

#### rs-fMRI preprocessing and functional connectome construction

The rs-fMRI data were minimally preprocessed following the HCP pipeline, including gradient distortion correction, motion correction, spatial normalization to Montreal Neurological Institute (MNI) space, and artifact removal using ICA-FIX.^55^ Additional preprocessing was performed using the Data Processing & Analysis for Brain Imaging (DPABI) toolbox.^52^ Nuisance signals, including white matter and cerebrospinal fluid signals, were regressed out, without global signal regression. The data were then band-pass filtered (0.01–0.1 Hz). To construct individual functional connectomes, the cerebral cortex was parcellated using the Schaefer 400-region functional atlas.^53^ Pearson correlation coefficients between regional time series were computed and Fisher *Z*-transformed, resulting in a symmetric 400 × 400 functional connectivity matrix for each subject.

#### tb-fMRI preprocessing and task activation estimation

The tb-fMRI data were resampled onto HCP grayordinates space and minimally smoothed using a 2-mm full-width at half-maximum (FWHM) kernel. MSMAll registration was applied to improve functional and structural alignment,^56^ followed by additional smoothing (4-mm FWHM). Task-evoked activation was estimated using a standard general linear model, resulting in 47 contrast maps across seven paradigms per participant. One representative contrast was selected for each task paradigm: Language Processing vs. Arithmetic (Language − Math), Theory of Mind vs. Random (ToM − Random), 2-back vs. 0-back, Faces vs. Shapes, Cue vs. Baseline, Reward vs. Punishment, and Relational Matching vs. Control Matching. Vertex-wise activation values were projected onto the Schaefer 400-region atlas, and mean activation within each region was extracted.

#### Connectome-constrained predictive modeling and NACI calculation

To quantify the extent to which intrinsic functional connectivity constrains task-evoked activation, a connectome-constrained predictive model based on the activity flow framework was implemented. Predicted activation in region *i* was estimated as a weighted sum of activations from all other regions:

$$\hat{d_{i}}=\frac{1}{N_{i}}\sum_{j=1,j\neq i}^{N_{i}}d_{j}A_{ij}$$

where *N*_*i*_ denotes the number of regions (here, *N*_*i*_ = 399), *d*_*j*_ represents observed activation in region *j*, and *A*_*ij*_ denotes resting-state functional connectivity between regions *i* and *j.* Both positive and negative connectivity weights were retained. The Neural Activation Constraint Index (NACI) was defined as the Pearson correlation between predicted and observed activation patterns across all cortical regions.

#### Spectral clustering

Each subject was represented by a seven-dimensional NACI vector, with each dimension corresponding to one of the seven task paradigms, resulting in a 1,005 × 7 feature matrix. Spectral clustering was performed directly on the NACI feature matrix by constructing a similarity matrix across subjects using a Gaussian kernel. To determine the optimal number of clusters, spectral clustering was conducted across candidate cluster numbers from K = 2 to K = 6. For each candidate K, the mean silhouette coefficient was calculated to quantify inter-cluster separation and intra-cluster cohesion. The optimal cluster number was defined as the K with the highest mean silhouette coefficient.

#### Robustness analyses

To evaluate the robustness of NACI, supplementary analyses were conducted using four alternative analytical strategies: (1) recomputing functional connectivity with global signal regression, (2) restricting analyses to positive connectivity weights only, (3) using an alternative parcellation scheme based on the HCP multimodal parcellation (Glasser-360),^54^ and (4) applying a moderate connectivity threshold (FC > 0.1) to define neighbors. The spectral clustering procedure described above was repeated under each of these strategies. Stability of NACI was assessed using Pearson correlations, and subgroup agreement was evaluated using consistency rates and Cohen’s kappa.

### Quantification and statistical analysis

#### Statistical analysis

First, differences in NACI across the seven task paradigms were assessed using repeated-measures analysis of variance (ANOVA), followed by post hoc paired t-tests to characterize pairwise differences between task paradigms. Second, NACI and demographic variables were compared between the spectral clustering-defined groups using independent-sample t-tests for continuous variables and chi-squared tests for categorical variables. Behavioral variables, including cognitive functions and task performance, were compared between groups using general linear models with age and sex included as covariates. Third, Pearson correlation analyses were performed to examine the associations between NACI and cognitive functions or task performance across all subjects. Statistical significance was set at two-sided *p* < 0.05 after false discovery rate (FDR) correction for multiple comparisons. All statistical analyses were conducted using SPSS Statistics (version 26.0; IBM Corp., Armonk, NY), MATLAB (R2021a; MathWorks, Natick, MA), R (version 4.1.0; R Foundation for Statistical Computing, Vienna, Austria), and Python (version 3.8; Python Software Foundation).

#### Sensitivity analyses

To assess whether group differences in cognitive functions and task performance were influenced by potential confounding variables, general linear models were used to compare the high- and low-constraint groups. Age and sex were included as covariates in the main analysis. Sensitivity analyses were then performed by further adjusting for head motion, years of education, and annual household income. *F*-values indicate the main effect of group, and *p*-values were corrected for multiple comparisons using false discovery rate (FDR) correction.

## Supplemental information

Document S1. Figures S1 and S2 and Tables S1–S4

Table S5. Full robustness analysis results across all conditions
