# Supplementary material for: Neurobiological and behavioral relevance of intrinsic functional connectome constraints on task-evoked neural activation
Source: iScience. 2026 Jul 22;29(8):116903. doi: 10.1016/j.isci.2026.116903 (PMC13523837; doi:10.1016/j.isci.2026.116903)
Supplement: Supplementary file 1 [file mmc1.pdf]

iScience, Volume *29*

Supplemental information

Neurobiological and behavioral relevance of intrinsic functional connectome constraints on task-evoked neural activation

Chao Tao, Shunshun Cui, Yuhao Shen, Yan Cheng, Jiajia Zhu, and Yongqiang Yu

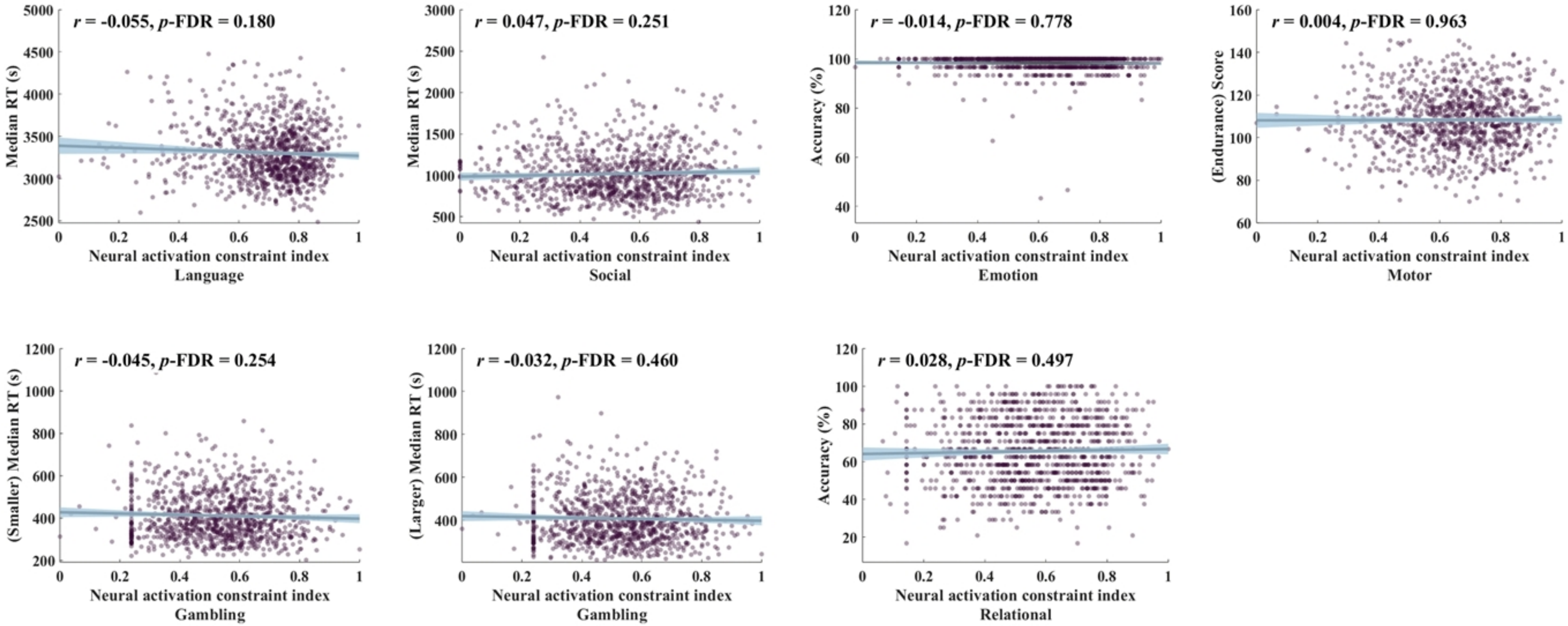

Supplementary Figure 1. Scatter plots displaying the non-significant correlations between NACI from several task paradigms and their corresponding task performance. Abbreviations: NACI, Neural Activation Constraint Index; RT, reaction time; FDR, false discovery rate.

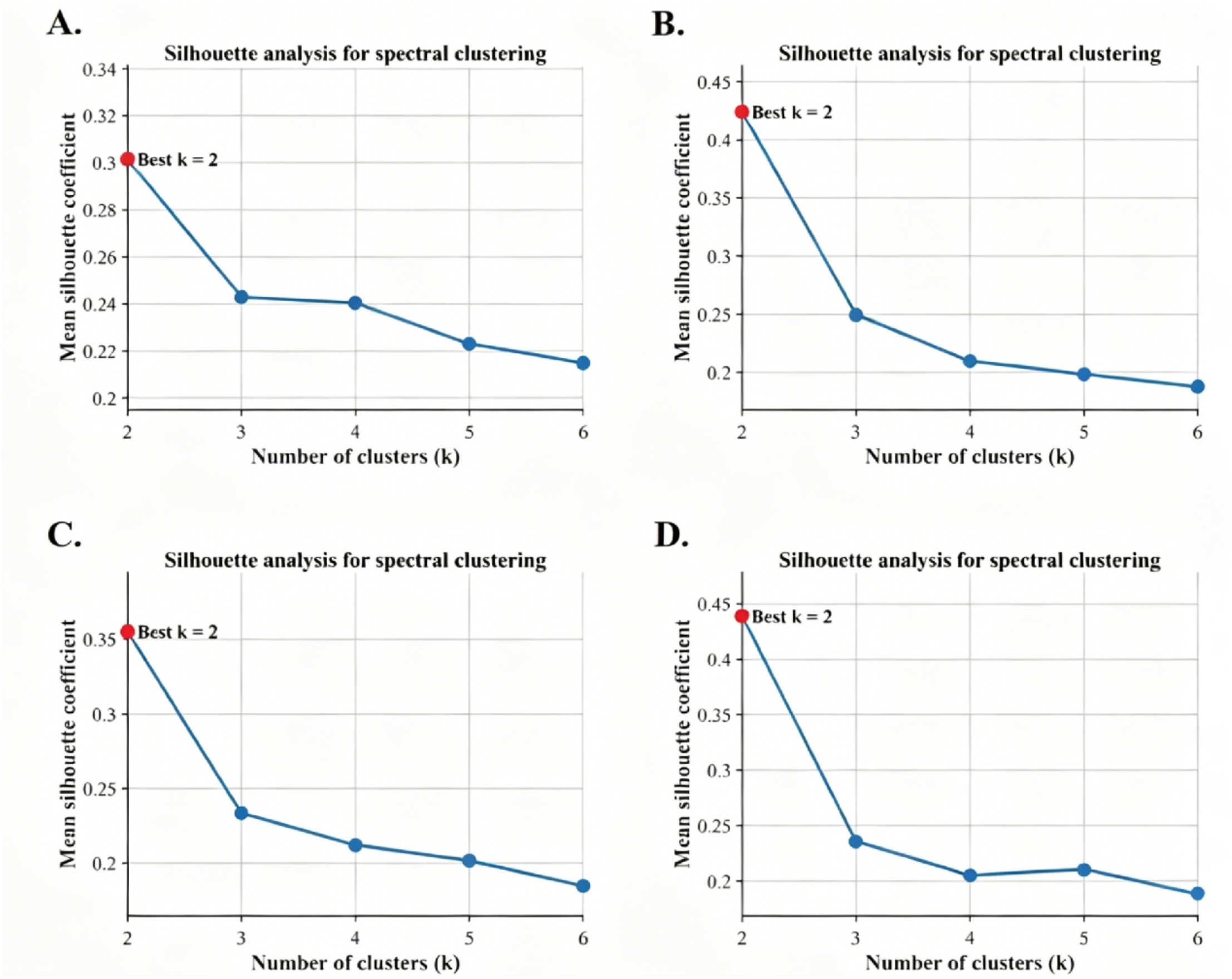

Supplementary Figure 2. Robustness of cluster-number determination across alternative analytical strategies. Silhouette Coefficient curves were generated for spectral clustering across candidate cluster numbers from $K = 2$ to $K = 6$ under three robustness conditions: (A) recomputing functional connectivity with global signal regression, (B) restricting analyses to positive connectivity only, and (C) using an alternative parcellation scheme based on the HCP multimodal parcellation (Glasser-360), and (D) applying a connectivity threshold (FC > 0.1) to define functionally connected neighbors.

Supplementary Table 1. Results of the paired comparisons in NACI across the seven tasks

	t-value	*p*-FDR
Language vs Social	41.110	<0.001
Language vs Working memory	21.482	<0.001
Language vs Emotion	40.829	<0.001
Language vs Motor	30.569	<0.001
Language vs Gambling	62.406	<0.001
Language vs Relational	39.822	<0.001
Social vs Working memory	-14.492	<0.001
Social vs Emotion	-1.468	0.143
Social vs Motor	-12.765	<0.001
Social vs Gambling	19.246	<0.001
Social vs Relational	1.574	0.122
Working memory vs Emotion	13.243	<0.001
Working memory vs Motor	3.732	<0.001
Working memory vs Gambling	33.290	<0.001
Working memory vs Relational	15.627	<0.001
Emotion vs Motor	-12.381	<0.001
Emotion vs Gambling	20.707	<0.001
Emotion vs Relational	2.999	0.003
Motor vs Gambling	34.647	<0.001
Motor vs Relational	14.488	<0.001
Gambling vs Relational	-16.105	<0.001

Abbreviations: NACI, Neural Activation Constraint Index; FDR, false discovery rate.

Supplementary Table 2. Comparisons in cognitive functions and task performance between the high and low constraint groups

Variable	High constraint group	Low constraint group	*F*-value	*p-FDR*
CardSort	103.21±9.63	101.48±10.01	9.277	0.003
ReadEng	108.35±14.40	105.72±15.05	10.093	0.002
PicSeq	105.69±15.85	104.52±17.43	0.288	0.592
Flanker	102.53±10.03	100.95±9.70	8.099	0.006
PicVocab	110.97±14.85	107.21±15.22	19.189	<0.001
ProcSpeed	105.76±19.37	101.15±20.37	12.944	0.001
ListSort	104.03±13.54	102.63±12.84	4.376	0.046
CogFluidComp	107.55±16.74,	103.58±17.23	13.616	<0.001
CogEarlyComp	108.52±15.45	104.94±16.07	13.577	<0.001
CogTotalComp	116.02±19.76	110.56±20.23	20.166	<0.001
CogCrystalComp	111.73±16.40	108.28±17.22	13.632	<0.001
Language Task Story Acc	94.64±8.33	93.62±8.50	3.876	0.049
Language Task Story Median RT	3277.85±307.17	3353.38±343.07	11.955	<0.001
Social Task Median RT TOM	993.52±278.26	1058.94±307.97	11.325	<0.001

Working memory Task 2bk Acc	85.24±9.88	81.35±11.22	38.384	<0.001
Working memory Task 2bk Median RT	958.01±144.57	973.72±138.71	2.534	0.128
Emotion Task Face Acc	98.49±3.08	98.12±4.55	2.366	0.136
Emotion Task Face Median RT	775.35±124.15	805.96±140.67	14.934	<0.001
Motor (Endurance)	108.82±13.67	107.44±14.00	8.771	0.004
Motor (Dexterity)	101.32±9.62	98.95±9.76	9.798	0.002
Gambling Task Median RT Larger	402.40±108.36	415.19±118.94	4.870	0.037
Gambling Task Median RT Smaller	406.44±107.74	420.49±120.42	5.559	0.026
Relational Task Rel Acc	67.65±17.55	62.00±17.49	22.888	<0.001
Relational Task Rel Median RT	1985.78±410.30	2005.34±440.33	0.395	0.553

Abbreviations: CardSort, Dimensional Change Card Sort; ReadEng, Oral Reading Recognition; PicSeq, Picture Sequence Memory; Flanker, Flanker Inhibitory Control and Attention; PicVocab, Picture Vocabulary; ProcSpeed, Pattern Comparison Processing Speed; ListSort, List Sorting Working Memory; CogFluidComp, Cognitive Fluid Intelligence Composite; CogEarlyComp, Cognitive Early Childhood Composite; CogTotalComp, Cognitive Total Composite; CogCrystalComp, Cognitive Crystallized Intelligence Composite; Acc, accuracy; RT, reaction time; FDR, false discovery rate.

Supplementary Table 3. Sensitivity analysis of group differences in cognitive functions and task performance after additional covariate adjustment.

Variable	*F*-value	*p*-FDR
CardSort	3.321	0.069
ReadEng	0.598	0.459
PicSeq	0.666	0.459
Flanker	4.974	0.069
PicVocab	5.626	0.061
ProcSpeed	8.345	0.016
ListSort	0.606	0.459
CogFluidComp	4.785	0.069
CogEarlyComp	3.311	0.138
CogTotalComp	5.371	0.062
CogCrystalComp	1.800	0.270
Language Task Story Acc	1.389	0.318
Language Task Story Median RT	11.071	0.007
Social Task Median RT TOM	9.578	0.010
Working memory Task 2bk Acc	20.148	<0.001
Working memory Task 2bk Median RT	1.628	0.286
Emotion Task Face Acc	1.104	0.352
Emotion Task Face Median RT	11.530	0.007
Motor (Endurance)	0.283	0.595
Motor (Dexterity)	2.596	0.172
Gambling Task Median RT Larger	2.724	0.170
Gambling Task Median RT Smaller	2.941	0.160
Relational Task Rel Acc	10.027	0.010
Relational Task Rel Median RT	1.243	0.335

Abbreviations: CardSort, Dimensional Change Card Sort; ReadEng, Oral Reading

Recognition; PicSeq, Picture Sequence Memory; Flanker, Flanker Inhibitory Control and Attention; PicVocab, Picture Vocabulary; ProcSpeed, Pattern Comparison Processing Speed; ListSort, List Sorting Working Memory; CogFluidComp, Cognitive Fluid Intelligence Composite; CogEarlyComp, Cognitive Early Childhood Composite; CogTotalComp, Cognitive Total Composite; CogCrystalComp, Cognitive Crystallized Intelligence Composite; Acc, accuracy; RT, reaction time; FDR, false discovery rate.

Supplementary Table 4. Correlations between NACI from the seven tasks and cognitive functions

	Language	Social	Working memory	Emotion	Motor	Gambling	Relational
CardSort	0.092	0.008	0.145	0.037	0.059	0.057	0.070
	(*p*-FDR=0.013)	(*p*-FDR=0.898)	(*p*-FDR<0.001)	(*p*-FDR=0.412)	(*p*-FDR=0.150)	(*p*-FDR=0.164)	(*p*-FDR=0.097)
ReadEng	0.097	0.017	0.169	-0.018	0.043	0.059	0.064
	(*p*-FDR=0.009)	(*p*-FDR=0.730)	(*p*-FDR<0.001)	(*p*-FDR=0.730)	(*p*-FDR=0.312)	(*p*-FDR=0.160)	(*p*-FDR=0.122)
PicSeq	0.049	-0.027	0.131	-0.014	0.036	0.036	0.040
	(*p*-FDR=0.233)	(*p*-FDR=0.573)	(*p*-FDR<0.001)	(*p*-FDR=0.783)	(*p*-FDR=0.416)	(*p*-FDR=0.443)	(*p*-FDR=0.375)
Flanker	0.056	0.022	0.124	0.037	0.001	0.023	0.097
	(*p*-FDR=0.160)	(*p*-FDR=0.641)	(*p*-FDR<0.001)	(*p*-FDR=0.412)	(*p*-FDR=0.996)	(*p*-FDR=0.641)	(*p*-FDR=0.009)
PicVocab	0.149	0.014	0.194	0.027	0.057	0.068	0.003
	(*p*-FDR<0.001)	(*p*-FDR=0.783)	(*p*-FDR<0.001)	(*p*-FDR=0.573)	(*p*-FDR=0.160)	(*p*-FDR=0.105)	(*p*-FDR=0.965)
ProcSpeed	0.097	0.017	0.158	0.023	0.073	-0.030	0.060
	(*p*-FDR=0.009)	(*p*-FDR=0.730)	(*p*-FDR<0.001)	(*p*-FDR=0.641)	(*p*-FDR=0.071)	(*p*-FDR=0.533)	(*p*-FDR=0.150)
ListSort	0.051	0.001	0.138	0.020	0.005	0.011	-0.006

	(*p*-FDR=0.208)	(*p*-FDR=0.996)	(*p*-FDR<0.001)	(*p*-FDR=0.685)	(*p*-FDR=0.932)	(*p*-FDR=0.838)	(*p*-FDR=0.932)
CogFluidComp	0.103	0.006	0.215	0.030	0.060	0.023	0.078
	(*p*-FDR=0.006)	(*p*-FDR=0.932)	(*p*-FDR<0.001)	(*p*-FDR=0.533)	(*p*-FDR=0.149)	(*p*-FDR=0,641)	(*p*-FDR=0.057)
CogEarlyComp	0.114	0.001	0.222	0.031	0.057	0.063	0.069
	(*p*-FDR=0.002)	(*p*-FDR=0.996)	(*p*-FDR<0.001)	(*p*-FDR=0.512)	(*p*-FDR=0.160)	(*p*-FDR=0.145)	(*p*-FDR=0.100)
CogTotalComp	0.136	0.014	0.245	0.025	0.056	0.055	0.070
	(*p*-FDR<0.001)	(*p*-FDR=0.783)	(*p*-FDR<0.001)	(*p*-FDR=0.601)	(*p*-FDR=0.160)	(*p*-FDR=0.186)	(*p*-FDR=0.097)
CogCrystalComp	0.125	0.011	0.185	0.004	0.037	0.069	0.029
	(*p*-FDR<0.001)	(*p*-FDR=0.838)	(*p*-FDR<0.001)	(*p*-FDR=0.956)	(*p*-FDR=0.412)	(*p*-FDR=0.105)	(*p*-FDR=0.620)

Abbreviations: NACI, Neural Activation Constraint Index; CardSort, Dimensional Change Card Sort; ReadEng, Oral Reading Recognition; PicSeq, Picture Sequence Memory; Flanker, Flanker Inhibitory Control and Attention; PicVocab, Picture Vocabulary; ProcSpeed, Pattern Comparison Processing Speed; ListSort, List Sorting Working Memory; CogFluidComp, Cognitive Fluid Intelligence Composite ; CogEarlyComp, Cognitive Early Childhood Composite; CogTotalComp, Cognitive Total Composite; CogCrystalComp, Cognitive Crystallized Intelligence Composite; FDR, false discovery rate.
